# Herd-Level Mastitis-Associated Costs on Canadian Dairy Farms

**DOI:** 10.3389/fvets.2018.00100

**Published:** 2018-05-14

**Authors:** Mahjoob Aghamohammadi, Denis Haine, David F. Kelton, Herman W. Barkema, Henk Hogeveen, Gregory P. Keefe, Simon Dufour

**Affiliations:** ^1^Faculté de Médecine Vétérinaire, Université de Montréal, St-Hyacinthe, QC, Canada; ^2^Canadian Bovine Mastitis and Milk Quality Research Network, St-Hyacinthe, QC, Canada; ^3^Ontario Veterinary College, University of Guelph, Guelph, ON, Canada; ^4^Faculty of Veterinary Medicine, University of Calgary, Calgary, AB, Canada; ^5^Wageningen University and Research Center, Wageningen, Netherlands; ^6^Atlantic Veterinary College, University of Prince Edward Island, Charlottetown, PE, Canada

**Keywords:** Cattle, dairy, mastitis, economic, Canada

## Abstract

Mastitis imposes considerable and recurring economic losses on the dairy industry worldwide. The main objective of this study was to estimate herd-level costs incurred by expenditures and production losses associated with mastitis on Canadian dairy farms in 2015, based on producer reports. Previously, published mastitis economic frameworks were used to develop an economic model with the most important cost components. Components investigated were divided between clinical mastitis (CM), subclinical mastitis (SCM), and other costs components (i.e., preventive measures and product quality). A questionnaire was mailed to 374 dairy producers randomly selected from the (Canadian National Dairy Study 2015) to collect data on these costs components, and 145 dairy producers returned a completed questionnaire. For each herd, costs due to the different mastitis-related components were computed by applying the values reported by the dairy producer to the developed economic model. Then, for each herd, a proportion of the costs attributable to a specific component was computed by dividing absolute costs for this component by total herd mastitis-related costs. Median self-reported CM incidence was 19 cases/100 cow-year and mean self-reported bulk milk somatic cell count was 184,000 cells/mL. Most producers reported using post-milking teat disinfection (97%) and dry cow therapy (93%), and a substantial proportion of producers reported using pre-milking teat disinfection (79%) and wearing gloves during milking (77%). Mastitis costs were substantial (662 CAD per milking cow per year for a typical Canadian dairy farm), with a large portion of the costs (48%) being attributed to SCM, and 34 and 15% due to CM and implementation of preventive measures, respectively. For SCM, the two most important cost components were the subsequent milk yield reduction and culling (72 and 25% of SCM costs, respectively). For CM, first, second, and third most important cost components were culling (48% of CM costs), milk yield reduction following the CM events (34%), and discarded milk (11%), respectively. This study is the first since 1990 to investigate costs of mastitis in Canada. The model developed in the current study can be used to compute mastitis costs at the herd and national level in Canada.

## 1. Introduction

Mastitis imposes considerable economic losses on the dairy industry all over the world. This economic burden is due to the additional expenditures on mastitis prevention and treatment, and to the losses due to various factors including reduced milk production, culling and discarded milk. Altogether, these mastitis-related expenditures and production losses constitute the basic components of the mastitis economic model proposed by Halasa et al. (1). For instance, cows affected with mastitis may produce less milk and infected quarters may produce poor quality or even inconsumable milk that needs to be discarded. Furthermore, clinical mastitis (CM) needs to be detected and treated by the farm personnel, which requires time and drugs, and will result in further discarded milk due to drug withdrawal time. Sometimes, more complicated mastitis issues may require the intervention of a veterinarian. In case the infection does not respond well to the treatment, or in case of chronic untreatable and contagious infections, the cow may be culled from the herd and replaced by a healthy cow. In addition, preventive measures are increasingly adopted by producers to help improve udder health during both lactation and dry periods (2).

In the framework proposed by Halasa et al. (1), reduction in milk production following CM or due to subclinical inflammation is an important component of the costs. Therefore, many studies have developed models to estimate the impact of high SCC or of pathogen-specific CM on subsequent milk production (3–5). These models are of great help for estimating the amount of milk not produced following mastitis and associated costs, and could certainly be used in different countries and settings.

However, other components (e.g., costs of drugs, labor, materials and investments) may vary among countries and are influenced by factors such as resource prices, cost of milk production, policies controlling the dairy market, and producers’ preferences for adopting prevention measures. For these reasons, studies on mastitis cost estimation should be conducted based on source populations restricted to a single geographical region such as a country or even a state (6–8).

Only one study investigated some of the mastitis-related expenditures on Canadian dairy farms (9). In this study, adoption proportion, cost, and efficiency of the practices included in the five-point plan to control contagious mastitis were investigated (10). Since the nineties, many other udder health-related practices have become common practices on Canadian dairies. Moreover, some expenses typical of modern dairy farms were not included in the initial economic model proposed by Halasa et al. (1). For example, over 80% of Canadian dairy producers participate in regular dairy herd improvement (DHI) programs. Since DHI programs are commonly used for udder health monitoring using SCC measurements, this expense should, perhaps, also be considered as a mastitis cost. However, it is not clear whether producers would still use these programs if it was not from mastitis (i.e., can we consider that participation in a DHI program is strictly a mastitis-related expense?).

The objectives of the current study were to estimate herd-level costs of mastitis on Canadian dairies and to investigate how these costs are distributed across the different costs components using an economic model derived from the Halasa et al. (1) model. This study is the first part of a project aiming at investigating mastitis costs at the national level and to describe costs fluctuations over time.

## 2. Materials and Methods

The research protocol was submitted to the University of Montreal ethics committee (Comité d’éthique de la recherche en santé, CERES) for evaluation. After reviewing the documents, the committee concluded that the project did not need an ethic approval for research with human participants since the dairy producers involved provided data but were not themselves targeted by this research (and thus did not meet the definition of a human participant).

### 2.1. Economic Framework

For the current study, the mastitis economic framework proposed by Halasa et al. (1) was used as a foundation. A cross-sectional study (described below) was designed to collect data on factors previously identified in the latter study to have an impact on mastitis costs. Selected factors were those associated with current expenditures for mastitis treatment and control and mastitis-associated output losses (e.g., culling, discarded milk, reduced milk yield) which could be readily estimated by dairy producers, and included factors related to: drugs, discarded milk, veterinary services, labor, product quality, diagnostic, culling, materials and investments. Among components in the framework proposed by Halasa et al. (1), the increased risk of other diseases following CM was not included in this study since the causal association between CM and other health problems is not well demonstrated and reverse causation cannot be excluded. Although several studies provided evidence for effects of mastitis on reproductive efficiency (11–13), no consensus was found among these studies regarding its precise effect and subsequent economic impacts. For each cost component, equations were formulated to estimate the cost over a year for a given herd. Details regarding computation of the different costs components are given in the following sections. An exhaustive list of the equations used is presented as Table S1 in  Appendix A. Beyond these equations describing how costs were related to the various farm data, some broader assumptions also had to be make. Whenever such a broad assumption was made, it will be explicitly identified in the text as “assumption #”.

#### 2.1.1. Milk Yield Reduction

Reduced milk yield following a CM case was estimated using the results of the study by Seegers et al. (7) reporting that a cow experiencing CM produced 5% less milk in her whole lactation (regardless of parity, isolated pathogen, and new versus repeated nature of the CM case). In order to estimate the overall milk production loss due to subclinical mastitis (SCM) the model suggested by Fetrow et al. (14) was used. In this model, a reduction of 190 kg of milk per milking cow is assumed for every 1 unit increase in the herd average linear score.

#### 2.1.2. Drugs

Because of concerns regarding economic efficiency of SCM treatment during lactation and risk of antimicrobial residues (15, 16), treatment of cows with SCM during the milking period is infrequently done in Canada. Therefore, apart from dry cow treatment, we assumed that drugs were not used for the treatment of SCM during the lactation (assumption #1). Furthermore, therapeutic protocols are often selected based on severity of clinical signs, and on many farms not all CM cases are treated. Different treatment protocols were, therefore, considered to be used for mild and moderate CM (i.e., abnormal milk with or without abnormal quarter appearance, but without systemic signs) compared to severe CM (i.e., systemic clinical signs) (17). Mild and moderate CM, when treated, were assumed to be treated solely with intramammary antimicrobials (assumption #2). For treatment of severe CM, producers commonly also systemically treat with antimicrobials and anti-inflammatory drugs in addition to the typical intramammary treatment. We assumed that the most common treatment for severe CM would consist, in addition to local treatment, of a 3-d administration of systemic antimicrobials plus 1 dose of a nonsteroidal anti-inflammatory drug (assumption #3).

Consequently, to estimate costs for drugs used for CM treatment, we took into account for each farm the number of CM cases over a year, the proportion of severe cases, the proportion of mild and moderate CM cases that were treated, the mean number of days a case is treated, the frequency of drug administrations per day, and drugs’ costs per administration.

In addition to drugs used for CM treatment, intramammary antimicrobial infusions are generally administered to all quarters of all cows at drying off. The cost for these later drugs was included in the materials and investments section (see below).

#### 2.1.3. Discarded Milk

On a dairy farm, milk may be discarded because of mastitis for 3 different reasons: (1) following treatment of CM (due to drug withdrawal time); (2) following CM cases that are not treated, but for which milk still has to be discarded until return of its normal appearance; or (3) in high SCC herds to manage bulk milk SCC (BMSCC) by diverting (i.e., discarding) milk of high SCC cows from the bulk tank. In the current study, these 3 sources of discarded milk were considered.

Milk production in Canada is regulated by a milk supply management system, and leasing milk quota to another producer is not permitted for most dairies. Therefore, producers have to find a way to fulfil their quota despite the discarded milk. This can be achieved by increasing production of cows or by keeping more cows in order to maintain the amount of milk shipped. The last option for the farmer is to sell some quota. In the current study, we assumed that dairy producers keep more cows than needed to fill their quota to cope up with the discarded milk and milk yield reduction (assumption #4). Consequently, the extra costs associated with the discarded milk and the milk yield reduction are the costs for having the same amount of milk produced by another cow, rather than the market value of the milk (1).

In some situations, however, the discarded milk is used as another input on the dairy, mainly to feed calves (18). This practice should be discouraged due to concerns about calves’ health (19–21), but using the discarded milk instead of a milk replacer helps mitigate some of the losses due to the discarded milk and is still often used. However, in case other readily available inputs are sufficient to feed calves (e.g., fresh cows’ milk), then no additional value can be returned from the discarded milk. In the current study, whenever milk discarded following CM was reported to be used to feed the calves, the money saved on milk replacer was deducted from the discarded milk costs. Milk discarded due to high SCC from cows with apparently normal milk was considered to be entirely used to feed calves (assumption #5) and the money saved on milk replacer was, therefore, deducted from the discarded milk costs.

In the current study, to estimate amount of discarded milk for treated CM, we took into account the proportion of CM cases in the herd that received treatment, treatment duration, drugs withdrawal time (obtained from the drug labels), and average daily milk production. Whenever a producer reported using more than 1 treatment regimens, mean treatment duration, mean withdrawal time, and mean treatment costs were used for CM cases occurring on this farm. Regarding non-treated CM cases, average time interval between CM diagnosis and time the milk from the animal was returned to the bulk tank was used instead of drug withdrawal time. Finally, amount of discarded milk for managing the BMSCC was estimated using the number of cow-days of production discarded. For each source of discarded milk, the costs of the discarded milk could be estimated using the production costs associated with having that volume of milk produced by other cows.

#### 2.1.4. Veterinary Services

In some cases, a veterinarian is consulted regarding a, usually severe, CM case. To estimate costs for this component, the number of CM cases for which a veterinarian consultation was sought, and the average cost for a veterinary visit (excluding the drug costs) were taken into account. Dairy producers also spend money to get professional advice concerning udder health issues, which is a cost over and above treatment of a CM case (e.g., routine monitoring, outbreak investigation, high SCC problems). Amount spent on such professional advice was, therefore, also considered in the current economic model.

#### 2.1.5. Labor

To estimate costs of labor associated with mastitis treatment, the average time spent working on a CM case (for diagnosis, initial treatment, follow-up treatment and separate milking), and the hourly wages were taken into account. Note that, as previously mentioned, we assumed that SCM does not result in any additional treatments during the lactation (see assumption #1); hence, no labor costs were associated with SCM treatment. However, time spent for applying various preventive measures (e.g., pre- and post-milking teat disinfection, dry cow treatment) were considered in the current study. For these later costs, we assumed 1 s per teat for pre- and post-milking teat disinfection, and 2 min per cow for administration of dry cow treatment [based on (22); assumption #6].

#### 2.1.6. Product Quality

Additional costs associated with product quality may occur because of premium loss or penalty payment for high BMSCC. Contamination with antimicrobial residues is another factor threatening product quality. In addition to penalty paid and premium lost, cost of insurance for milk quality (e.g., to cover antimicrobial residues or high BMSCC problem) paid was also taken into account for this component. Since premium program of milk quality varies among provinces, provincial milk boards were contacted to collect information on premium program of each province (e.g., threshold for high quality milk, premium payment value), but producers were directly surveyed for information on penalty and insurance payments.

 The effect of milk quality on cheese yield, shelf-life and consumers’ complaints were considered to mainly influence the milk processing companies, not dairy farms (assumption #7); therefore, these costs were not included in current calculations.

#### 2.1.7. Diagnostics

Producers may collect milk samples from cows having CM or SCM. To estimate costs associated with diagnostics, total number of samples collected in a year for CM and SCM, apart from regular DHI tests, and analysis costs per samples were taken into account. In addition, it was not clear whether DHI costs should be considered as mastitis-associated expenses (due to the SCC measurements). We inquired with both Canadian DHI companies and producers to figure out whether DHI participation costs should be included in diagnostic costs or not.

#### 2.1.8. Culling and Mortality

When a primiparous cow is culled or dies, costs incurred can be assumed to be those of rearing or buying an equivalent first lactation cow minus any money received for meat or milk sale. When a multiparous cow is culled or dies, the difference in milk production between the culled and replacement cow (assuming the replacement cow is a primiparous cow) was added to these costs. However, when a cow dies on farm from CM, no money is received in exchange for meat or milk sale. Furthermore, in this latter case expenditures for carcass disposal have to be considered.

Costs for primiparous cows that were culled or died were estimated using the number of first lactation cows which were culled due to CM or SCM or died due to CM, the costs for rearing or buying a replacement first lactation cow, the money received for meat or milk sale, and, for dead primiparous cows, the costs for carcass disposal.

When estimating costs for replacing a multiparous cow, these same factors were taken into account. In addition, the fact that a mature cow produces 1.3 times more milk than a first lactation was considered (23). In the current study, we assumed that no cow died from SCM (assumption #8).

#### 2.1.9. Materials and Investments

Among expenditures for mastitis prevention measures, only those performed exclusively for mastitis prevention were taken into account. These included pre- and post-milking teat disinfection, use of gloves for milking, dry cow therapy, and mastitis vaccination. Other measures such as milking machine maintenance, towels, bedding and floor management, manure collection, and other measures used for environmental hygiene were not accounted for, since these measures would still have to be used if it was not from mastitis, or are implemented to control a range of diseases such as lameness, gastrointestinal infections, etc.

### 2.2. Data Collection Tools

All variables needed for the economic model are listed in Table 1. The main data collection tool used to collect information on mastitis-associated expenditures in Canadian dairy herds was a questionnaire consisting of 35 open-ended and multiple-choice questions. The questionnaire was first developed in English and then translated to French. The English version of the questionnaire is available as Table S2 in Appendix B. The questionnaire was mailed in January 2016 to the 374 dairy producers participating in the second phase of the Canadian National Dairy Study [CNDS (25); (26)]. This latter study is similar to the National Animal Health Monitoring Study (NAHMS dairy) conducted every 7 years in the United States (27). In the CNDS, an initial general survey was sent to all Canadian registered dairy farms, and 1,193 producers completed this first survey with response rate of 11% (26). In that initial survey, participants were asked if they were willing to participate in a phase two study involving answering additional questionnaires and on farm visits. Among the initial respondents, 825 agreed to participate in the phase 2 study and a sample of 374 dairy farms was selected for the second phase of the CNDS. The 374 farms were selected so the proportion of producers by province and of DHI-participating herds reflected the official records from the provincial dairy boards (British Columbia, *n* = 20; Alberta, *n* = 20; Saskatchewan, *n* = 10; Manitoba, *n* = 10; Ontario, *n* = 133; Québec, *n* = 121; New-Brunswick, *n* = 17, Nova-Scotia, *n* = 18; Prince Edward Island, *n* = 20, and Newfoundland; *n* = 5). A questionnaire was sent by mail, in the language of communication previously indicated by the dairy producer in the phase 1 of the CNDS. A 10 Canadian dollar (CAD) gift card incentive was provided for completing the questionnaire.

**Table 1:** Variables used to estimate costs for mastitis-related expenditures, culling, and discarded milk.

Component	Required variables
General information	Number of milking cows
Milk yield reduction	Mean BMSCC, number of milking cows, costs of production of 1 kg of milk*, Number of CM cases, cow mean daily milk production
Drug	Number of CM cases, proportion of CM cases that were severe^*†*^, proportion of moderate and mild CM cases that received treatments, type of drugs used, frequency of administration and duration of treatment, price per drug unit^*‡*^
Discarded milk	Number of CM cases, proportion of CM cases that received treatment, average duration of treatment, withdrawal time of used drugs^*§*^, duration of discarding milk in CM cases that are not treated, number of cow-days of discarding milk to manage BMSCC, mean cow daily milk production, costs of production of 1 kg of milk*, proportion of discarded milk fed to calves, price milk replacer^*¶*^
Veterinary services	Number of CM cases, proportion of CM cases visited by a veterinarian, average cost for a veterinary visit, expenses on professional advices regarding herd mastitis issues
Labor	Number of CM cases, average time spent working on a CM case, average hourly wage**
Product quality	Cost of insurance, amount paid in penalties, premium loss
Diagnostic	Number of samples collected in a year for CM and SCM apart from regular DHI tests, costs per sample
Culling and mortality	Number of first lactation and older cows which were culled or died due to CM or SCM, costs for rearing or buying a first lactation cow, difference in milk production between primiparous and multiparous^*†*^^*†*^, money received for meat or milk when selling a cow, money spent on carcass disposal for dead cows
Materials and investments (Material and labor for implementing preventive measures)	Expenses for pre- and post-milking teat disinfection, gloves used for milking, dry cow therapy, and mastitis vaccination, required labor time for implementing pre- and post-milking teat disinfection and dry cow therapy^*‡*^^*‡*^

All values were reported for last 12 months (i.e., year 2015). Unless specified otherwise, source of information for variables was the producers’ questionnaire sent to the 374 Canadian National Dairy Study participants.

*Source: (Canadian Dairy Commission 2015 cost of production study http://www.cdc-ccl.gc.ca/CDC/userfiles/file/REPORT_-_P&E_-_2015_COP_Indexed_to_Q1_2016_Booklet_-_July_2016.pdf.

^†^Source: Canadian Bovine Mastitis Research Network National Cohort of Dairy Farm study (24)

^‡^Source: suggested retail price of the largest Canadian veterinary drug distributor (CDMV) St-Hyacinthe, QC, Canada, https://www.cdmv.com/en/veterinary-boutique.sn

^§^Source: drug labels

^¶^Source: mean retail price of the 5 most popular brands

**Source: Statistics Canada http://www5.statcan.gc.ca/cansim/pickchoisir?lang=eng&p2=33&id=2810035#F19

^†^^†^Friggens et al., (1999) (23).

^‡^^‡^van Soest et al., (2016) (22).

To estimate CM incidence, dairy producers were asked about the number of CM cases on their farm in the last 12 months. In the questionnaire, a CM case was defined as a cow producing abnormal milk (flakes, watery…) with or without a swollen udder, fever or loss of appetite. Subclinical mastitis was also referred to as “elevated SCC”. In the general section of the questionnaire, questions on the number of milking cows, average production per cow per day, and mean BMSCC were included. The questionnaire is available in Appendix B.

The costs of the intramammary treatments reported to be used were based on retail prices suggested by the largest Canadian veterinary drug distributor (CDMV, St-Hyacinthe, QC). For producers who reported using more than 1 type of intramammary drugs to treat CM, we used the mean price of the various treatments reported to be used.

In our economic model, severe CM cases were deemed to receive systemic treatment in addition to intramammary treatment. To determine proportion of CM cases being severe, the database of the Canadian Bovine Mastitis and Milk Quality Research Network’s National Cohort of Dairy Farms was consulted (24). In short, in this cohort the cows from 91 farms were followed in 2007 and 2008, and all CM events as well as severity of these events were recorded on a 1 to 3 scale as described by Sears et al. (17). In this study, a severity score of 3 (i.e., severe CM) was observed in 20% of CM cases when using the 74 herds validated for CM reporting (28). We therefore assumed that 20% of the CM cases reported by dairy producers would be severe cases and treated with both local and systemic treatments. In severe cases, additional costs due to systemic antimicrobial (3 doses of 9.6 g of trimethoprim/sulfamethoxazole) and anti-inflammatory (1 single dose of 1.3 g of flunixin meglumine) injections were estimated at 25.40 CAD using, again, retail prices suggested by CDMV.

Costs for production of 1 kg of milk was obtained from the Cost of Production Study (2015) conducted by Canadian Dairy Commission (Ottawa, Ontario). Using this later study conducted on a sample of 240 dairy farms, cost of production was established at 0.78 CAD/kg of milk. Regarding costs of milk replacer, the retail prices of the 5 most popular brands of milk replacer were obtained through internet resources and phone calls to distributors. Taking into consideration the mixing directions for each brand, a mean price of 0.49 CAD/litre (range: 0.42, 0.62 CAD) of reconstituted milk replacer was obtained and used as a fixed value.

Finally, based on labor wages used in the Cost of Production Study and obtained from Statistics Canada (25), wages for dairy personnel (most often the owner and its family on Canadian dairy farms) were fixed at 34.50 CAD/h (29).

### 2.3. Data Management and Statistical Analyses

All returned questionnaires were coded and entered in a database (Access 2016, Microsoft Corp., Redmond, WA). Specific codes for missing, not applicable, and unreadable responses were used. The database was then transferred to SAS 9.4 (SAS Institute Inc., Cary, NC) for computation of indices and descriptive statistics. For each quantitative variable, minimum, SD, first quartile, mean, median, third quartile, and maximum were calculated. Unlikely values were identified. and impossible responses were excluded from calculation. The distribution of each variable was depicted to evaluate the normality of the distribution.

Then, for each herd, expenses due to the different mastitis-related components were computed by applying the values reported by the dairy producer to the equations reported in Appendix A. On a few occasions one of the producer’s answer was incomplete and precluded computation of expenses, in these cases the median observed value was used instead. For instance, a few producers reported having culled cows due to mastitis, but did not report the price received for culled cows. For these the median price for culled cows observed in the dataset was used.

Expenses that could be attributed to either CM or SCM were summed separately. All expenses were then divided by the number of milking cows and multiplied by 100 to report cost/100 milking cows. Clinical mastitis-related expenses were also reported as CAD/CM case by dividing total amount spent for CM-related expenses by number of CM cases reported. Again, simple descriptive statistics (minimum, SD, first quartile, mean, median, third quartile, and maximum) were then computed for each standardized cost component.

For each herd, proportion of the costs attributable to a specific component was also computed by dividing absolute costs for this component by total herd mastitis-related expenditures. Proportion of the mastitis-related expenses due to CM, SCM, and other expenditures were computed in a similar manner.

## 3. Results

**Figure 1:**
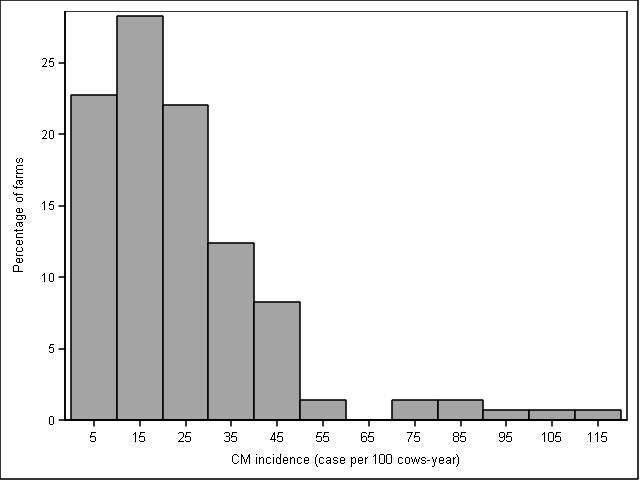
Distribution of clinical mastitis incidence (in CM cases per 100 cow-year) in 2015 based on producers’ reports in a sample of 145 Canadian dairy producers.

**Figure 2:**
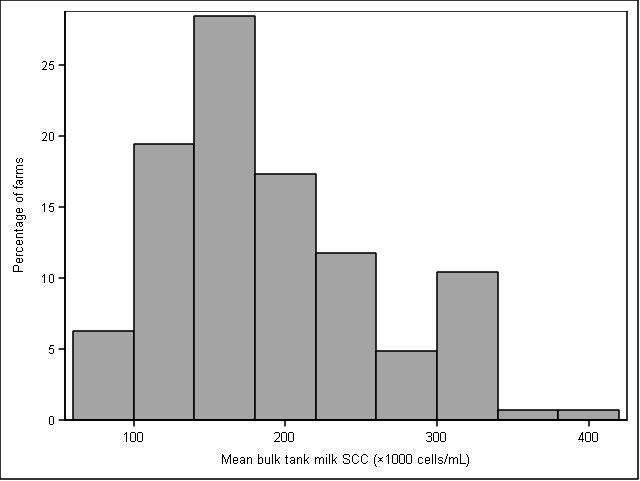
Distribution of mean 2015 bulk milk SCC in a sample of 145 Canadian dairy producers.

Between January and May 2016 145 producers responded to the questionnaire (39% response rate). Median number of milking cows was 60 (range: 20 to 550 cows) with an average milk production of 32 kg/day (SD: 5.7 kg). Median self-reported incidence of CM was 19 cases/100 cow-year (Q1 and Q3 of 11 and 31 cases/100 cow-year, respectively; Figure 1). Mean self-reported BMSCC was 184,000 cells/mL (SD: 69,000 cells/mL; Figure 2), and 67% of respondents participated in DHI.

Adoption of various mastitis-preventive measures is presented in Table 2. Most producers reported using post-milking teat disinfection (97%) and dry cow therapy (93%), and a substantial proportion of producers reported using pre-milking teat disinfection (79%) and wearing gloves during milking (77%). Using vaccination for preventing mastitis was used by a minority of producers (35%). Distribution of mastitis costs attributable to CM, SCM, materials and investment, and product quality are presented in Table 3 and are discussed for each costs component in the following sections.

**Table 2:** Adoption proportion of various mastitis-preventive measures in 2015 in a sample of 145 Canadian dairy producers.

Prevention measure	Adoption proportion (%)	95% Confidence interval (%)
Pre-milking teat disinfection	79	73–83
Post-milking teat disinfection	97	94–99
Dry cow therapy	93	89–97
Wearing gloves at milking	77	70–84
Use of mastitis vaccines	35	27–43

**Table 3:** Herd distribution of mastitis-related costs (in CAD per 100 cows/year) in 2015 in a sample of 145 Canadian dairy producers.

Component	N missing	Min	Percentiles			Max	Mean	SD
			25th	50th	75th			
Clinical Mastitis								
Milk yield reduction	0	0	4,213	6,703	10,773	41,632	8,483	8,357
Drugs	3	0	131	349	694	5,908	508	638
Discarded milk	3	0	817	1,445	2,580	12,007	2,096	1,960
Veterinary services	5	0	0	0	161	3,396	155	393
Labor	2	0	310	657	1,294	9,554	1,185	1,643
Diagnosis	17	0	0	59	226	3,378	187	381
Culling and mortality	36	0	4,605	9,037	17,222	61,304	14,045	14,045
Total clinical mastitis		0	13,372	19,889	33,439	94,253	27,752	19,830
Subclinical mastitis								
Milk yield reduction	1	0	17,928	24,110	32,217	47,057	24,461	10,041
Discarded milk	8	0	0	87	548	10,150	532	1,280
Veterinary services	0	0	0	0	0	9,375	266	1,112
Diagnosis	17	0	0	0	217	7,500	231	733
Culling	23	0	3,229	8,571	15,600	58,585	11,653	12,401
Total subclinical mastitis		2,345	24,162	34,859	46,405	98,381	37,048	18,027
Materials and investments								
(Prevention measures)								
Materials pre-milking teat disinfection	0	0	200	969	1,585	7,619	1,193	1,251
Labor pre-milking teat disinfection	0	0	2,758	2,758	2,758	2,758	2,187	1,121
Materials post-milking teat disinfection	0	0	920	1,500	2,610	6,714	1,937	1,452
Labor post-milking teat disinfection	0	0	2,758	2,758	2,758	2,758	2,606	632
Materials dry cow therapy	0	0	943	1,683	2,500	16,667	1,837	1,740
Labor dry cow therapy	0	0	91	91	91	91	81	28
Gloves	0	0	24	156	386	1,800	251	283
Vaccines	0	0	0	0	571	4,650	422	836
Total Prevention measures		0	8,106	10,477	13,134	24,495	10,515	4,236
Product quality								
Insurance	52	0	0	0	105	2,857	133	381
Penalty	0	0	0	0	0	3,759	35	325
Premium loss	0	0	0	0	1,164	11,534	1,394	2,791
Total product quality		0	0	0	2,843	11,912	1,564	2,828
Total		16,508	51,014	66,178	93,634	182,581	76,657	35,400

N missing, number of producers with ≥1 missing answer for figures needed to compute a cost component and for which median value for that figure had to be used for computation; Min, minimum; Max, maximum.

### 3.1. Milk Yield Reduction

Median economic value of milk yield reduction following CM cases was estimated at 6,703 CAD per 100 cows-year (range: 0 to 41,632; Table 3). Median economic value of milk yield reduction due to SCM was estimated at 24,110 CAD per 100 cows-year (range:0 to 47,057; Table 3).

### 3.2. Drugs

Ten (7%) producers indicated that their farms were certified organic. Median proportion of CM cases that were treated in all herds including both organic and commercial was 90%. Most producers used intramammary infusion solely, with treatment duration ranging from 1 to 9 d. Median cost for treatment of mild or moderate CM was 21 CAD and median cost for drugs for severe CM was 46 CAD. Total drug expenditure for treatment of CM was estimated at 349 CAD per 100 cows-year (range: 0 to 5,908).

One interesting finding regarding drugs used for CM treatment is that producers often treated cows longer than the labelled treatment regimen. Seventy producers reported 1 single treatment used for their typical mild or moderate CM case, so their treatment protocols could be compared to the labelled drug regimen. Among these producers, only 12 (17%) reported using the labelled treatment protocol. Among the 58 (83%) producers using off-label treatments, 2 (4%) treated for 1.5 d with a product labelled for a 2-d treatment, and 54 (93%) treated for longer than the label recommended (mean: +2 d; range: 0.5 to 6 d). A total of 14 (24%) producers used the drugs with higher administration frequency (i.e., twice a day administration of a product labelled for once a day administration), and 3 (5%) producers reported using drugs with a lower administration frequency (i.e., once a day administration of a product labelled to be administered twice a day).

### 3.3. Discarded Milk

The median period milk was discarded in case of CM treatment was 6 d (range: 4 to 12 d), which included treatment days plus drug withdrawal time; whereas, in cows with untreated CM the median duration of discarding milk was 2 d (range: 0–21 d). Most producers reported using a substantial proportion of milk discarded due to CM to feed calves (median: 25% of discarded milk; range: 0 to 100%). Median cost of discarded milk due to both treated and untreated CM after subtracting the value of wasted milk fed to calves was 1,445 CAD per 100 cow-year (range: 0 to 12,007; Table 3), and median cost of discarded milk for 1 CM case was 79 CAD/per CM case (range: 2 to 686).

Among participating producers, 41% reported discarding milk of cows with high SCC. Overall median number of cows per year for which milk was discarded was 1 cow per yr (range: 0 to 37) and the milk of these cows was discarded on average during 7 d (range: 0 to 100). Amount of discarded milk due to SCM was not significantly associated with the BMSCC (i.e., low and high SCC herds equally discarded milk due to SCM). Median costs of discarded milk for high SCC cows were estimated at 87 CAD per 100 cow-year (range: 0 to 10,150 CAD; Table 3).

### 3.4. Veterinary Services

Producers reported calling a veterinarian for less than 1% of CM cases (range: 0 to 75% of CM cases) and median cost for a veterinary visit (excluding drugs) was 100 CAD. Consequently, median veterinary cost for CM cases were 0 CAD per 100 cow-year (range: 0 to 3,396 CAD; Table 3).

In addition, only 24% of producers reported having used a veterinarian for udder health monitoring, high SCC, or CM outbreak investigation in the last 12 months. Median costs for veterinary services for such monitoring or investigation was, therefore, estimated at 0 CAD per 100 cows-year (range: 0 to 9,375; Table 3).

### 3.5. Labor

Median time working on a CM case (for diagnosis, initial treatment, follow-up treatment and separate milking) was 1 h (range: 0 to 8.5 h). Median expenditures for extra labor due to CM was estimated at 657 CAD per 100 cow-year (range: 0 to 9,554; Table 3), and 34 CAD per CM case (range: 3 to 239).

### 3.6. Product Quality

Having to pay a penalty for high BMSCC milk is relatively uncommon in Canada. Nevertheless, among our respondents, 3 producers reported paying penalties (one of 100 CAD, one of 500 CAD, and the last one 5,000 CAD) within 12 months. Median costs for penalty were, therefore, estimated at 0 CAD per 100 cow-year (range: 0 to 3,759 CAD; Table 3). Proportion of respondents who had insurance coverage for antimicrobial residues in milk was 66%; however, many respondents did not know the exact portion of their insurance payment being specifically for milk quality insurance. The median annual costs for insurance was 0 CAD per 100 cow-year (range: 0 to 2,857; Table 3).

Milk quality premium system varied among provinces. In some provinces (Ontario, Newfoundland and Labrador, Nova Scotia), producers did not receive bonus for milk quality, so premium loss was considered zero for herds located in these provinces. In New Brunswick, there was no per hectoliter premium system. Instead, the offered premium on milk quality was a yearly cash awards to the 10 producers who had the best milk quality results. Therefore, premium loss was not considered for herds in New Brunswick.

In the Western provinces (Alberta, British Columbia, Manitoba, Saskatchewan), a premium was paid to herds with average BMSCC ≤250,000 cells/mL, whereas, in Quebec and Prince Edward Island the threshold was BMSCC ≤200,000 cells/mL. Mean value of premium in western provinces, Quebec, and Prince Edward Island was 0.28, 0.50, and 0.25 CAD/hl respectively. Moreover, in Quebec there was an additional 0.29 CAD/hl premium for herds with BMSCC ≤150,000 cells/mL.

Because many herds were located in provinces were no milk quality premiums were distributed, and because many of the herds in provinces having milk quality premiums did get that premiums, losing a premium for milk quality was an uncommon event (*n* = 37 herds). Median estimated value for premium loss was 0 CAD per 100 cows-year (range: 0 to 11,534).

### 3.7. Diagnosis

The proportion of herds reporting collecting and analyzing (sent to the laboratory or analyzed on farm) milk samples from CM cows was 66%. Median expenditures for diagnosis of CM were 59 CAD per 100 cow-year (range: 0 to 3,378 CAD; Table 3). Fifty percent of producers reported submitting milk samples from cows suspected of SCM for bacteriological culture, and median costs of 0 CAD per 100 cow-year (range: 0 to 7,500 CAD; Table 3) were observed for SCM diagnosis.

Based on producers’ responses, main motivation of most producers (82%) for participation in DHI program was not monitoring cows’ SCC, and most reported that they would still pay for DHI participation even without any SCC information. Therefore, although 68% of herds were participating in DHI programs with a median frequency of 10 herd tests per year, membership fees for DHI programs were not considered as a mastitis-associated expenditure and were excluded from our calculations.

### 3.8. Culling and Mortality

Among respondents, 54 and 17% reported having culled or lost, respectively, first lactation cows due to CM in the last 12 months. Median number of culled and dead heifers due to CM were respectively of 0 (range: 0 to 23) and 0 animal per 100 cow-year (range: 0 to 12). Median cost for 1 culled heifer was 1,350 CAD. Median cost for culled heifers was 0 CAD per 100 cow-year (range: 0 to 46,154 CAD). Median costs attributable to heifers dying from CM were 0 CAD per 100 cow-year (range: 0 to 31,800 CAD).

A total of 86 and 39% of respondents, respectively, reported having culled or lost ≥2 nd lactation cows due to CM in the last 12 months. Median number of culled and dead cows due to CM were 3 (range: 0 to 21) and 0 animals per 100 cow-year (range: 0 to 9), respectively. Median cost for culling 1 cow was 2,150 CAD per culled cow. Median costs attributable to culling ≥2nd lactation cows were 5,911 CAD per 100 cow-year (range: 0 to 58, 585 CAD). Median costs for ≥2nd lactation cows dying from CM were 0 CAD per 100 cow-year (range: 0 to 33,913 CAD). Consequently, median costs associated with culling and mortality of heifers and mature cows were 9,037 CAD per 100 cow-year (range: 0 to 61,304 CAD; Table 3).

A total of 47 and 84% of dairy producers reported having culled heifers and cows, respectively, due to SCM in the last 12 months. Median number of heifers culled for SCM was 0 animals per 100 cow-year (range: 0 to 23 animals). Median number of cows culled for SCM was 4 animals per 100 cow-year (range: 0 to 22 animals). Median costs for culling heifers due to SCM in a 100-cows herd was 0 CAD (range: 0 to 46,154 CAD). Median costs for culling adult cows due to SCM in a 100 cows herd was 6,743 CAD (range: 0 to 58,585 CAD). Median total costs due to culling of heifers and cows due to SCM was 8,571 CAD (range: 0 to 58,585 CAD; Table 3).

### 3.9. Material and Investment

Median costs of prevention measures are indicated in Table 3. In terms of materials and labor, the 3 most expensive preventive measures were pre- and post-milking teat disinfection and dry cow therapy.

### 3.10. Relative Costs

**Figure 3:**
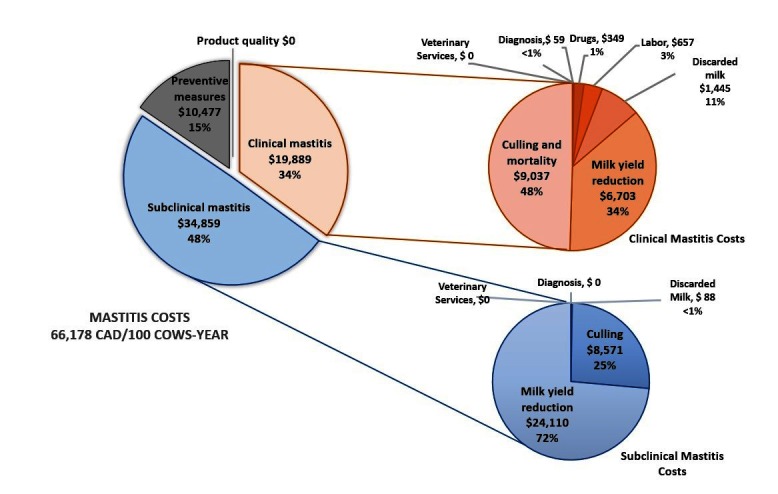
Absolute values and relative importance (in %) of the different cost-components for the median herd in Canada (100 cows-year)

Median expenses for a CM case were 744 CAD per CM case (range: 50 to 5,349 CAD). Median estimated costs were 13,487 CAD per 100 cow-year for CM, and 34,344 per 100 cow-year for SCM (Table 3). Relative importance of the different cost-components for the median herd is presented in Figure 3. Overall SCM (48%) was the costliest category, followed by CM (34%), and materials and investment (mainly for applying preventive measures; 15%). In the median herd, most of CM costs were due to culling and mortality (48%) and then milk yield reduction (34%; Figure 3). Regarding SCM, most of the costs (72%) were due to milk yield reduction and 25% were due to culling (Figure 3).

## 4. Discussion

The objective of this study was restricted to describing the current costs of mastitis on Canadian dairy farms and the distribution among the different costs components. The aim of this study was to give a broad picture of these costs and, therefore, some components with a lower relative importance were not included in our calculations. For example, the potential negative effects of mastitis on cows’ reproductive performances or risk of other diseases were excluded from our calculation due to uncertainty about association between mastitis and these events, not mentioning the complexity inherent to estimating these impacts. Moreover, preventive measures implemented to control both mastitis and other diseases were excluded from mastitis costs. The estimated costs would have been higher if the potential effect of mastitis on subsequent health had been considered and if mastitis-related prevention measures that are not solely used for mastitis prevention had been included. Size of this bias is difficult to predict, but, given that disease events and reproductive inefficiency usually have substantial economic impacts on dairies, the bias is likely to be considerable.

The estimated median CM incidence in the current study (i.e., 19 cases per 100 cows-year) was close to previous estimates in Canadian dairies. In the study by Olde Riekerink et al. (30), mean incidence rate of CM was estimated at 23 cases per 100 cows-year during 2003 to 2005. In the National Cohort of Dairy Farms (NCDF) study, conducted in 2007 and 2008 a median incidence rate of 21.3 case per cow-305 days was reported (28). At first sight CM incidence rate may seem to be decreasing over time, but in Elghagguf et al. (28) only first cases were included, whereas in Olde Riekerink et al. (30) and the current study, both first and repeated cases were included. In addition, Elghafghuf et al. (28) study was prospective with frequent follow-ups and more precise measurements, while in the current study, number of CM cases within 12 months reported by producers were used to estimate the overall CM incidence, which may possibly result in a certain underestimation. Similarly, the mean self-reported BMSCC in the current study (184,000 cells/mL) was close to reports by prior studies in Canada such as the study by Olde Riekerink et al. (31) which reported that geometric mean BMSCC was 185,000 cells/mL.

Comparing the adoption level of preventive practices in the study of Gill et al. (9) with the results in current study shows that among those measures that were recommended in both years 1990 and 2015, the highest increase was evident in implementing dry cow therapy which has increased by 18%. Comparing adoption levels of preventive practices in the current study and those in the study by Olde Riekerink et al. (31) showed that practices less implemented in 2010 such as pre-milking teat disinfection, dry cow therapy, wearing gloves at milking, and using mastitis vaccines are more and more adopted by producers.

In the current study, mastitis costs appear to be substantial (662 CAD per cow per year for a typical Canadian dairy), with most of the costs (48%) being attributed to SCM (due mainly to costs attributable to the subsequent reduced milk yield), and 34 and 15% due to CM and implementation of preventive measures, respectively. Since there are no other recent equivalent studies on mastitis costs in Canada, or in other countries with a similar production system, it is difficult to directly compare these results to other studies. Nevertheless, in a study conducted by van Soest et al. (22), preventive measures were the most expensive cost component, estimated at € 120/cow-year and representing 50% of total mastitis costs. In that same study, the next most important component was milk yield reduction (€ 69/cow-year; 29% of costs), followed by culling (€ 20/cow-year; 8%) and discarded milk (€ 20/cow-year; also 8% of costs). In the current study, the estimated costs of milk yield reduction (313 CAD/cow-year) and culling were higher (192 CAD/cow-year) than costs of preventive measures (105 CAD/cow-year) and discarded milk (19 CAD/cow-year). In addition, costs of culling and discarded milk were not presented separately for CM and SCM in van Soest et al. (22). Preventive measures can hardly be separated between CM and SCM since many of these measures are targeting both forms of the disease. Nonetheless, by dividing culling and discarded milk costs between CM and SCM, we were able to demonstrate in the current study that CM and SCM contribute almost equally to culling costs Additionally, although discarding milk for SCM is a relatively common practice applied by Canadian dairy producers, the amount of milk discarded for this reason is small compared to that of CM.

### 4.1. Clinical Mastitis Costs

Regarding CM costs in the current study, highest relative costs were due to culling and mortality (48%), milk yield reduction (34%), then discarded milk (11%), and, finally, labor (3%). In a Dutch study conducted when the Netherlands still had a supply management system for dairy (32), milk yield reduction and culling were identified as the two most substantial CM cost components with almost equal mean costs (€ 23/cow-year and € 22/cow-year, respectively). These two components were followed by cost of discarded milk (€ 9/cow-year) and then drugs (€ 6/cow-year). However, the 3 components of CM costs with highest values in the current study were in order culling, milk yield reduction, and discarded milk. Differences in relative importance of CM cost components in these two studies could be due to considerable differences in inputs such as costs of culling per cow and frequency of culling in CM cases. Moreover, in the current study additional costs of replacing culled multiparous cows by heifers due to their differences in milk production was taken into account. In contrast, in Huijps et al. (32) a fixed value (€ 480) was used as costs of culling a cow regardless of the cow parity. Moreover, in the current study the losses in future cow production associated with premature culling were not taken into account, resulting in an underestimation of the true culling costs.

 Typically, on Canadian dairy farms, most of the day-to-day work, including CM treatment, is conducted by the owners. Therefore, wages proposed by Statistics Canada and used to compute costs for CM treatment are quite high (34.5 CAD/h) compared to those of larger farms where employees would be paid to do that job. In a Finnish study, also conducted when there was a milk supply management system, after milk yield reduction (31%), veterinary services and drugs (24%), premature culling (23%), and then discarded milk (18%) had highest shares in total CM costs (33). A noticeable difference between our study and that of Heikkila et al. (33), is that in Finland, unlike Canada, only veterinarians are allowed to treat mastitis cases. Therefore, CM treatment was much more expensive in Finland than in Canada, where veterinarians are called for less than 1% of CM cases.

### 4.2. Subclinical Mastitis Costs

The only cost component for SCM that was measured specifically for SCM in the aforementioned studies was reduced milk production (22, 32). To our knowledge, other components such as culling and discarded milk were never presented separately for CM and SCM. It is, therefore, difficult to compare our results with those of previously published studies. In the current study, two most substantial cost components of SCM were milk yield reduction (72%) and culling (25%). Although costs of veterinary advices for SCM control were reported to be near zero (Figure 3), these costs are possibly underestimated by dairy producers since these veterinary consultations are often intertwined with other activities (e.g., reproduction, calve health) occurring during regular herd health visits.

### 4.3. Preventive measures costs

Van Soest et al. (22) estimated costs of preventive mastitis control measures on Canadian farms at € 120/cow-year), which was higher than costs of other important components such as milk yield reduction and culling. The main contributor to preventive costs was the required labor to implement practices. To investigate the sensitivity of our economical model to our initial assumptions regarding the required labor for implementing these practices, we computed median costs estimates for teat disinfection time varying from 0.75 to 1.25 s/quarters and for a dry cow treatment administration time of 5 min (compared to the assumed 2 min). The median costs for a 100 cows dairy were slightly affected by the chosen teat disinfection time value (from 64,799 to 67,557 CAD/100 cow-year, for teat disinfection time varying from 0.75 to 1.25 s/quarters, respectively). Increasing labor time required for dry cow therapy to 5 min had almost no impact on the median mastitis costs (+136 CAD).

Moreover, it is noteworthy that preventive measures considered by van Soest et al. (22) were less mastitis-specific and included practices that are not performed exclusively for mastitis control (e.g., cleaning alley ways, cleaning cubicles). Whereas, by considering practices used exclusively for mastitis (i.e., pre- and post-milking teat disinfection, dry cow treatment, wearing gloves during milking, and mastitis vaccines), as in the current study, application of preventive measures was, of course, less expensive (105 CAD/cow-year).

### 4.4. Potential Biases

Some factors in the current study may have led to an underestimation of mastitis costs. For estimating CM costs, a single milk production ratio (1.3: 1.0) between multiparous and primiparous cows was considered in our computations, whereas this ratio is for comparing second and first lactation cows. The milk production ratio of third to first lactation cows would actually be slightly higher (23). Furthermore, older cows (i.e. ≥3 lactations) have higher risk to die or to be culled following CM (34, 35). Therefore, a considerable proportion of mature cows that died or were culled because of mastitis were possibly cows with ≥3 lactations. Consequently, CM culling costs were possibly underestimated. In addition, most CM cases occur in early lactation (36) which is the time when the cow is producing the most. This fact was not taken into account in the current study, since mean milk production was used to compute amount of discarded milk, resulting, again, in an underestimation of CM discarded milk costs.

In the current study, we also considered that SCM cases were not treated during the lactation, while, actually, some producers certainly used this practice. Such an assumption possibly led to an underestimation of drugs costs and of milk discarded due to SCM. Nevertheless, treating cows during the lactation for SCM would be a rare event in Canada and the impact of that later assumption is likely to be small.

 Feeding calves with raw waste milk is demonstrated to have negative impacts on calf and herd health by increasing the risk of antimicrobial resistance and bacterial shedding in the environment (19–21). However, in the current study the negative economic consequences of this practice were not taken into account.

Number of cows culled due to CM or SCM, a very important component of mastitis costs, was reported by dairy producers using retrospective data. Culling decisions, however, are mostly taken based on more than 1 single reason (37). Therefore, depending on the producers’ considerations when answering that specific question, proportion of mastitis-culled cows may have been over- or underestimated. In this case, direction of bias is difficult to predict. However, we could hypothesize that dairy producers would more likely forget to complete some of their records regarding culling, than complete extra records. Thus, the number of cows reported to be culled because of mastitis is likely to be an underestimation, which may, in turn, compensate for the fact that, for some of these cows, mastitis was possibly a minor component in the culling decision (e.g., a 305 DIM, low producing, open cow with mastitis would likely eventually be culled without the mastitis event).

 Premium losses were determined based on mean BMSCC reported by producers solely. However, there were other criteria for milk quality to get entitled for premium payment (e.g., individual bacterial count) which were not available to the authors. Moreover, annual mean BMSCC was used for this purpose instead of monthly mean; consequently the estimated value of premium loss could be biased. Since median relative costs of product quality were estimated 0%, the mentioned biases had no considerable impact on overall costs.

In the future, the economic model developed in the current study could be applied to all Canadian dairy farms using retrospective demographic data available in DHI and previous mastitis knowledge obtained from the National Cohort of Dairy Farms (24) to compute mastitis costs in Canada and to monitor mastitis cost fluctuations over time.

## 5. Conclusions

Costs of mastitis on Canadian dairy farms was substantial with median costs of 662 CAD/cow-year. Among the different components, milk yield reduction was the most cost component (313 CAD/cow-year; 46%). Costs for culling and implementation of preventive measures were the second and third most important cost components, respectively.

## Author Contributions

All authors participated to planning of the study, MA, DH, and SD were responsible for the data collection and for the statistical analyses, MA and SD were responsible for drafting the manuscript, and all authors reviewed and contributed to the final manuscript.

## Conflict of Interest Statement

The authors declare that the research was conducted in the absence of any commercial or financial relationships that could be construed as a potential conflict of interest. As per the research agreement, aside from providing financial support, the funders have no role in the design and conduct of the studies, data collection and analysis or interpretation of the data. The researchers maintain independence in conducting their studies, own their data, and report the outcomes regardless of the results. The decision to publish the findings rests solely with the researchers.
