# Supplementary material for: Herd-Level Mastitis-Associated Costs on Canadian Dairy Farms
Source: Front Vet Sci. 2018 May 14;5:100. doi: 10.3389/fvets.2018.00100 (PMC5961536; doi:10.3389/fvets.2018.00100)
Supplement: Supplementary file 1 [file Table1.pdf]

APPENDIX A

Component	Equation	
Milk yield reduction	Eq. 1	Amount of herd milk loss due to CM = number of CM cases × 0.05[a] × mean cow daily milk production × 305[b]
	Eq. 2	Costs of milk loss due to CM = amount of herd milk loss due to CM × 0.78[c]
	Eq. 3	Average linear score of individual cow SCC in herd = (Log10(BMSCC) -1.6)/0.24[d]
	Eq. 4	Amount of milk loss due to SCM=number of milking cows×190[d]× (Average linear score of individual cow SCC in herd -1)
	Eq. 5	Costs of milk loss due to SCM = Amount of milk loss due to SCM×0.78[c]
Drug	Eq. 6	Cost of drugs for total local treatment= mean price of used intramammary infusions per quarter per day × days of treatment × frequency of treatments per day
	Eq. 7	Cost of drugs to treat **mild** to **moderate** CM = number of CM cases × 0.8[e] × proportion of CM cases receiving treatment × cost of drugs for total local treatment
	Eq. 8	Cost of drugs to treat **severe** CM = number of CM cases × 0.2[e] × proportion of CM cases receiving treatment × (cost of drugs for total local treatment + 25.4[f])
	Eq. 9	Total cost of CM treatment= Cost of drugs to treat **mild** to **moderate** CM + Cost of drugs to treat **severe** CM
Discarded milk	Eq. 10	Amount of milk discarded for **treated CM** = number of CM cases × Proportion of CM cases treated × (duration of treatment + mean withdrawal time) × average daily milk production per cow
	Eq. 11	Amount of milk discarded for **untreated CM** = Number of CM cases × (1- Proportion of CM cases treated) × number of days before milk is put back in bulk tank × average daily milk production per cow
	Eq. 12	Total amount of milk discarded in CM cases (treated + untreated) = Amount of milk discarded for **treated CM** + Amount of milk discarded for **untreated CM**
	Eq. 13	Economic impact of discarding milk in **CM** cases = (total amount of milk discarded in **CM** cases × 0.78[c]) – (% of discarded milk used to feed calves × total amount of milk discarded in **CM** × 0.49[g])
	Eq. 14	Amount of milk discarded for **SCM** = Number of cows excluded from bulk tank for high SCC × number of days of this exclusion × mean daily milk production per cow
	Eq.15	Economic value of discarding milk in **SCM cases** = (Amount of milk discarded for **SCM** × 0.78[c]) – (Amount of milk discarded for **SCM** ×0.49[g])
Veterinary services	Eq.16	Cost of veterinary services for **CM** = number of CM cases × proportion of CM cases for which veterinarian is called × average cost for a veterinary visit

Component	Equation	
	Eq.17	Cost of veterinary services for herd **SCC** management = total expenditures on professional advices about herd mastitis issue
Labor	Eq.18	Cost of labor to manage CM = number of CM cases × time spent working on a CM case (diagnostic, initial treatment, follow-up treatment, separate milking) ×34.5[i]
Product quality	Eq.19	Cost of milk quality = yearly payment for insurance in case there is an insurance coverage + penalty payment for exceeding SCC limit + premium loss for exceeding SCC limit
Diagnostic	Eq.20	Cost of diagnostic procedure for **CM** = number of samples collected in a year for CM × cost of each sample
	Eq.21	Cost of diagnostic procedure for **SCM** = number of samples collected in a year for SCM cases × cost of each sample
Culling and mortality	Eq.22	Economic value of **culling** of **1st** lactation cows for **CM** = Number of 1st lactation cows culled due to CM × (costs for rearing or buying a 1st lactation cow - money received for meat or milk sale)
	Eq.23	Economic value of **1st** lactation cows **dying** from **CM** = Number of 1st lactation cows that died due to CM × (costs for rearing or buying a 1st lactation cow + money spent for carcass disposal)
	Eq.24	Economic value of **culling** of **older** cows for **CM** = Number of older cows culled due to CM × (1.3[h] × costs for rearing or buying a 1st lactation cow - money received for meat or milk sale)
	Eq.25	Economic value of **older** cows **dying** from **CM** = Number of older cows that died due to CM × (1.3[h] × costs for rearing or buying a 1st lactation cow + money spent for carcass disposal)
	Eq.26	Economic value of **culling 1st** lactation cows due to **SCM** = Number of 1st lactation cows culled due to SCM × (costs for rearing or buying a 1st lactation cow - money received for meat or milk sale)
	Eq.27	Economic value of **culling older** cows due to **SCM** = Number of older cows culled due to SCM × (1.3[h] × costs for rearing or buying a 1st lactation cow - money received for meat or milk sale)
Materials and investments (with required labor)	Eq.28	Economic value of required labor for pre-milking teat disinfection in 12 months= number of milking cows× (4/3600) [j] × 2[k] ×365[l]× 34.5[i]
	Eq.29	Economic value of required labor for post-milking teat disinfection in 12 months= number of milking cows× (4/3600) [j] × 2[k] ×365[l]× 34.5[i]
	Eq.30	Economic value of required labor for dry cow therapy in 12 months= number of milking cows×0.8[m] × (2/60) [n]× 34.5[i]
	Eq.31	Cost of pre-milking teat disinfection in 12 months + Economic value of required labor for pre-milking teat disinfection + Cost of post-milking teat disinfection in 12 months + Economic value of required labor for post-milking teat disinfection + Cost of dry cow therapy in 12 months + Economic value of required labor for dry cow therapy

Component	Equation	
		+ Cost of gloves used during milking in 12 months + Cost of mastitis vaccine in 12 months

All costs were multiplied by 100 and divided by number of milking cows to report value for a herd of 100 milking cows. Values were mostly obtained from producers through questionnaires except for factors with superscripts explained in following footnotes:

[a] (Seegers et al., 2003b)

[b] Days in 1 lactation

[c] Cost of producing 1 litre of milk in 2015 in Canadian dollar (Canadian Dairy Commission, 2015)

[d] (Fetrow et al., 1988)

[e] Proportion of mild to moderate versus severe CM among all CM cases (database of NCDF)

[f] Minimum additional cost of treatment for severe CM cases was considered 3 doses of trimethoprim /sulfamethoxazole as systemic antimicrobial and 1 dose of flunixin meglumine as anti-inflammatory drug for a cow with average body weight

[g] Mean cost of 1 litre reconstituted milk replacers for calves based on mixing directions and cost of used brands

[h] Average ratio of second to first parity lactational curve coefficients (Friggens et al., 1999)

[i] Hourly wage (Statistics Canada, 2015)

[j] Required time in hours for disinfection of all teats of 1 cow

[k] Assumed number of herd milking times per 24 hours

[l] Number of days per year

[m] Proportion of cows which were dried off in a herd per year

[n] Required time in hours to administer dry cow therapy for 1 cow (van Soest et al., 2016)
