# Supplementary material for: Herd-Level Mastitis-Associated Costs on Canadian Dairy Farms
Source: Front Vet Sci. 2018 May 14;5:100. doi: 10.3389/fvets.2018.00100 (PMC5961536; doi:10.3389/fvets.2018.00100)
Supplement: Supplementary file 2 [file Table2.pdf]

APPENDIX B

Economic of mastitis questionnaire

General

1. How many milking cows do you currently have? _________________cows

2. What is the cow average daily milk production on your farm? __________kg/day

3. How much does it cost to rear or buy a first lactation cow? _________________$

4. Do you use milk replacer to feed the calves?

❍ No – if No, please proceed to question 5
❍ Yes
  4.a. What brand of milk placer did you use during last 12 months? ____________
  4.b. What are the costs per kilogram of milk replacer? _________$/Kg powder

5. During the last 12 months, how much did you spend on these items to prevent mastitis?

  5.a. Pre milking teat-disinfection solution _________________$/year
  5.b. Post milking teat-disinfection solution _________________$/year
  5.c. Dry cow therapy _________________$/year
  5.d. Milking machine maintenance (regular inspection by technician, replacing parts and liners) _________________$/year
  5.e. Gloves for milking personnel _________________$/year
  5.f. Towels for drying teats during milking _________________$/year
  5.g. Vaccine against mastitis (costs for vaccine and administration) _________________$/year

6. was your farm participating in Dairy Herd Improvement (DHI) control in last 12 months?
❍ No – if No, pleases proceed to question 7
❍ Yes
  6.a. How many controls in the last 12 months? __________controls / year
  6.b. If it was not from SCC measurements, would you still use DHI control?
    o No
    o Yes

Clinical mastitis

In the current questionnaire clinical mastitis is defined as: a cow with abnormal milk (flakes, watery…) with or without a swollen udder, fever, loss of appetite…

7. How many cases of clinical mastitis did you have in the last 12 months? _________CM cases

8. On your farm, what proportion of cows with clinical mastitis is treated with drugs?
_________%

9. What drugs are used to treat a typical clinical mastitis case on your farm?

Antibiotics	Anti-inflammatory (if any)
❍ 17900 special formula forte<br>❍ Cefa-lak<br>❍ Pirsue<br>❍ Spectramast LC<br>❍ Other: _______________	❍ Anafen<br>❍ Metacam<br>❍ Banamine, Flunixine, Cronyxin<br>❍ Dexamethazone<br>❍ Flucort<br>❍ Predef 2X<br>❍ Other: _____________

10. How many times per day and for how long is the antibiotic administered?

_________________times per day, for _________________days

11. What is the cost of your typical clinical mastitis treatment?
_________________$/ treatment (complete whole treatment)

12. How much time do the farm personnel spend working on a typical clinical mastitis case (initial and follow-up treatments, separate milking, etc)? ______________hours

13. For clinical mastitis cases that are not treated, on average for how many days do you discard the milk? _________________days

❍ Not Applicable

14. What proportion of discarded milk from cows with clinical mastitis is used to feed the calves? _________________%

15. In what proportion of clinical mastitis cases do you call a veterinarian?
_________________%

16. On average what are the costs of the veterinarian when called for a clinical mastitis case (without cost of drugs)? _________________$

17. How many milk samples were collected and analyzed (sent to the lab or analyzed on-farm) for clinical mastitis cases in the last 12 months?
_________________samples/year

18. How much do the laboratory tests cost? _________________$/test

19. How many 1st lactation and older cows were culled or died due to clinical mastitis in the last 12 months?

______1st lactation cows culled

______1st lactation cows died

______older cows culled

______older cows died

20. When culling a cow with clinical mastitis on average how much money is received for meat or milk sale? _________________$

21. How much money is spent on carcass disposal in case a cow dies from CM? _________________$

22. Do you have insurance coverage for antimicrobial residue in the bulk tank?

- ❍ Yes
- ❍ No

23. If yes, what is the cost of this insurance? _________________$/year

Somatic cell count

24. What was your average bulk milk somatic cell count in the last 12 months? _________________x 1,000 cells/ml

25. How much did you lose as penalty for exceeding the SCC limit in the last 12 months? _________________$

26. How many milk samples were collected and analyzed (sent to the lab or analyzed on-farm) for high SCC cows in the last 12 months? _________________samples/year

27. How many cows were excluded from bulk tank because of high SCC in last 12 months? _________________cows

28. For how many days on average did you exclude milk from these cows?
_________________days

29. Is there a premium in your province for milk quality?

- ❍ Yes
- ❍ No

30. How much did you spend in the last 12 months for professional advices concerning udder health issues different than treatment of a clinical mastitis case (e.g. routine monitoring, outbreak investigation, high SCC problems)?

___________$/an

31. How many 1st lactation and older cows were culled due to high SCC last year?
_____1st lactation

_____older cows

32. How much money was received for meat or milk sale for cows culled because of high SCC? _________________$/cow

Thank you for your time, in completing this questionnaire

Simon Dufour, DMV, Ph.D.
Professeur / Professor
Département de pathologie et microbiologie
Faculté de médecine vétérinaire, Université de Montréal

Directeur scientifique / Scientific Director
Réseau canadien de recherche sur la mammite bovine et la qualité du lait
Canadian Bovine Mastitis and Milk Quality Research Network

450 773-8521 ext. 8605
450 778-8128
simon.dufour@umontreal.ca

www.reseaumammite.org / www.mastitisnetwork.org
C.P. 5000 Saint-Hyacinthe (Québec) CANADA J2S 7C6

Réseau canadien de recherche sur la mammite bovine et la qualité du lait
Canadian Bovine Mastitis and Milk Quality Research Network
